# Development of a novel global rating scale for objective structured assessment of technical skills in an emergency medical simulation training

**DOI:** 10.1186/s12909-021-02580-4

**Published:** 2021-03-25

**Authors:** Andreas Zoller, Tobias Hölle, Martin Wepler, Peter Radermacher, Benedikt L. Nussbaum

**Affiliations:** 1 Medical Faculty, Ulm University, Ulm, Germany; 2 Institute of Anaesthesiological Pathophysiology and Process Engineering, University Hospital Ulm, Ulm, Germany; 3 Department of Anesthesiology and Intensive Care Medicine, University Hospital Ulm, Ulm, Germany

**Keywords:** Global rating scale, Technical skills, Objective structured assessment, Emergency medicine, Simulation

## Abstract

**Background:**

Medical simulation trainings lead to an improvement in patient care by increasing technical and non-technical skills, procedural confidence and medical knowledge. For structured simulation-based trainings, objective assessment tools are needed to evaluate the performance during simulation and the learning progress. In surgical education, objective structured assessment of technical skills (OSATS) are widely used and validated. However, in emergency medicine and anesthesia there is a lack of validated assessment tools for technical skills. Thus, the aim of the present study was to develop and validate a novel Global Rating Scale (GRS) for emergency medical simulation trainings.

**Methods:**

Following the development of the GRS, 12 teams of different experience in emergency medicine (4th year medical students, paramedics, emergency physicians) were involved in a pre-hospital emergency medicine simulation scenario and assessed by four independent raters. Subsequently, interrater reliability and construct validity of the GRS were analyzed. Moreover, the results of the GRS were cross-checked with a task specific check list. Data are presented as median (minimum; maximum).

**Results:**

The GRS consists of ten items each scored on a 5-point Likert scale yielding a maximum of 50 points. The median score achieved by novice teams was 22.75 points (17;30), while experts scored 39.00 points (32;47). The GRS overall scores significantly discriminated between student-guided teams and expert teams of emergency physicians (*p* = 0.005). Interrater reliability for the GRS was high with a Kendall’s coefficient of concordance W ranging from 0.64 to 0.90 in 9 of 10 items and 0.88 in the overall score.

**Conclusion:**

The GRS represents a promising novel tool to objectively assess technical skills in simulation training with high construct validity and interrater reliability in this pilot study.

## Background

Simulations are increasingly used to train emergency care providers [1–4]. They are educationally effective and create a controlled and safe training environment, as they place trainees in realistic settings, that provide immediate feedback about questions, decisions and actions [5, 6]. According to Goolsby et al. simulation training increases medical students’ procedural confidence by providing an experiential learning environment [7]. It also increases medical knowledge of providers and may uncover further knowledge gaps compared to less interactive instruction methods [8]. Simulation trainings allow the assessment of both technical as well as behavioral performances amongst medical students [5, 9]. While raising awareness for the importance of non-technical skills, simulation can help to enhance patient care and safety by developing a safety culture and reducing medical errors on both personal and systemic levels [10–12].

Despite the wide use of simulations as a teaching and assessment tool, in 2010 Kardong-Edgren et al. outlined a lack of reliable and valid instruments to measure simulation effectiveness [13]. Furthermore, many instruments are based on student self-reported evaluation [13, 14]. Several tools to evaluate non-technical skills exist (e.g. the Anaesthetist’s non-technical skills assessment (ANTS) [15] or the Mayo High Performance teamwork scale [16]. However, apart from the Queen’s simulation assessment tool (QSATS), which is specifically validated for resuscitation scenarios [17], no valid tool to assess a participant’s global technical skillset in various emergency medical care scenarios is available. In contrast, objective structured assessment of technical skills (OSATS) is widely used in surgery to evaluate progress in training, since Martin et al. introduced this tool in 1995 [18–22]. In the original OSATS three scoring systems, a global rating scale, a detailed task specific checklist and a pass/fail judgement were used [21]. Yet in later versions of the OSATS the pass/fail judgement was not used anymore [18].

As no valid tools exist for the evaluation of technical skills in emergency medical training, the aim of the present study was to develop and validate a modified OSATS tool, as those had proven valuable in measuring progress in training [19, 20, 22].

In combination with rating tools for non-technical skills, educators and instructors may get a more integrated view on the performance of participants in high-fidelity simulation training of emergency scenarios.

## Methods

An experienced group of pre-hospital emergency medical care providers and teachers of emergency medicine (Table 1) comprising qualified paramedics and emergency physicians designed a GRS applicable for several kinds of emergency medical scenarios in a multi-step approach: An initial draft of the GRS was developed by two members of the expert group. The selection of items for the assessment and treatment of critically ill patients to be included in the GRS was based on current standards and guidelines, standard emergency medicine textbooks, the experts’ real-life emergency experience and on their observations from their work as simulation instructors [23–27]. Subsequently, the first draft was tested several times in student emergency medical simulation trainings. Items of the GRS were edited with respect to content and feasibility in the light of the experiences from the ‘test’ simulations. Next, two more members of the expert group, who both were not involved in the initial drafting, further evaluated the GRS and were allowed to make additional adjustments. Again, after conducting several test-runs in different scenarios, the GRS was handed to a consultant anesthetist who was not involved in the development so far for final revision. The GRS is complemented by a Task specific checklist (TSC) solely for non-traumatic patients which was newly established in a similar process as the GRS.

**Table 1:** Characteristics of the expert group developing the GRS with respect to their (pre-)clinical and teaching experience

Specialty	Clinical Expertise in pre-hospital emergency care	Teaching expertise
Paramedic, medical student	nine years of experience as paramedic	paramedic instructor, tutor in emergency medical courses at university, medical simulation trainings, licensed instructor for simulation-based training and CRM
Paramedic, medical student	Five years of experience as paramedic	tutor in emergency medical courses at university, support of medical simulation-based training
Anesthetist	licensed emergency physician, four years of experience as emergency physician	medical simulation trainings, licensed instructor for simulation-based training and CRM, lectures of emergency medicine
Anesthetist	licensed emergency physician, nine years of experience as emergency physician	medical simulation trainings, lectures of emergency medicine
Consultant Anesthetist	licensed emergency physician, 28 years of experience as emergency physician	medical simulation trainings, lectures of emergency medicine

**Fig. 1:**
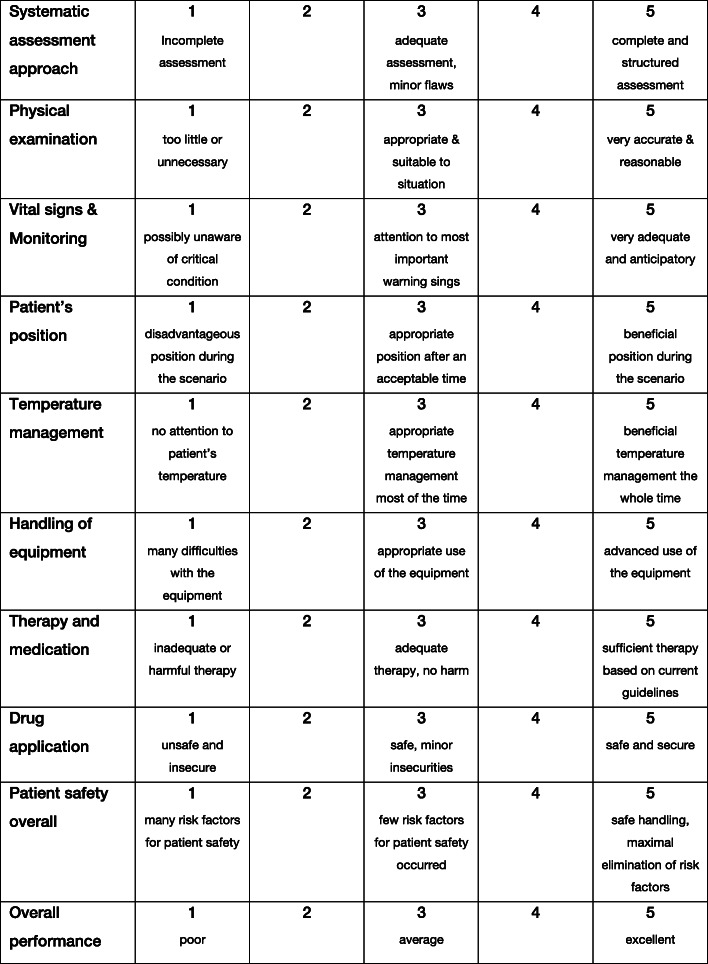
Global Rating Scale. The GRS consists of ten items, each rated on a five-point Likert scale. The maximum overall score is 50 points, minimum score is 10 points

**Fig. 2:**
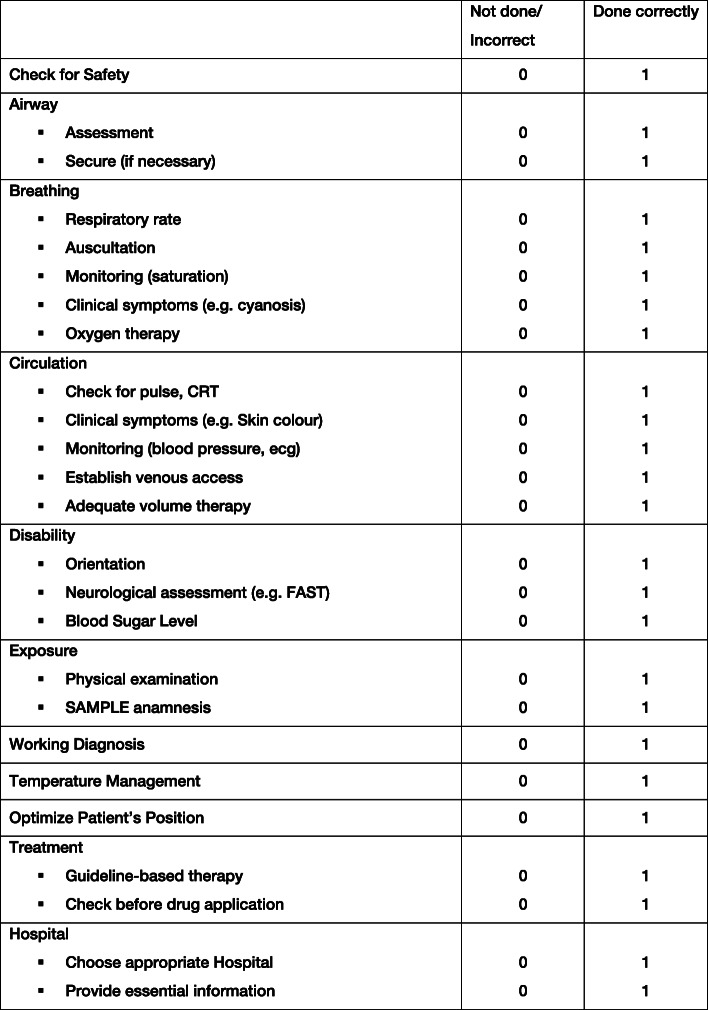
Task Specific Checklist. The Task Specific Checklist (TSC) complements the GRS, but is solely designed for non-traumatic patients. 25 items are either rated as ‘done correctly’ or ‘not done/incorrect’. CRT: capillary refill time, ecg: electrocardiogram, FAST: acronym meaning **F**ace **A**rms **S**peech **T**ime, SAMPLE: acronym meaning. **S**ymptoms, **A**llergies, **M**edication, **P**ast medical history, **L**ast oral meal, **E**vents

The GRS in the present study consists of 10 items incorporating a structured diagnostic approach, guideline conform therapy and patient safety aspects (Fig. 1). Each item is scored on a 5-point Likert scale resulting in an overall maximum score of 50 points and a minimum score of 10 points in the GRS. The TSC contains 25 items, which are either done correctly or not done/incorrect and therefore rated with 0 or 1 (Fig. 2).

For validation of the GRS, twelve emergency teams were compared in a pre-hospital high fidelity simulation scenario. In the simulation, a standardized patient with an injection pad to allow intravenous injection and drug application and a custom-made vest with built-in speakers to mimic pathologic lung sounds and heart murmurs was used. Further pathologies and vital signs were displayed by an ALSi simulation monitor from iSimulate. The emergency equipment was similar to common standards in prehospital care throughout Germany. The training scenario was identical for every team: a woman in her mid-fifties presenting with an acute coronary syndrome and a third-degree AV block. On scene, the patient was hemodynamically unstable presenting with dizziness, nausea and severe bradycardia (heart rate less than 40/min). According to the ERC bradycardia algorithm guideline-based therapy consisted either of administering epinephrine or external pacing [28].

Each team comprised two 4th year medical students (m = 4, f = 20) and a dedicated team leader defining the expert level of the team. The team leaders were either medical students (m = 2, f = 2) as well, certified EMTs/paramedics (m = 3, f = 1) or trained emergency physicians (m = 4) with experience in the field. All participants had to report their level of expertise and experience before team allocation. Team formation was aimed to ensure comparable levels of training within groups.

After obtaining informed written consent, all simulations were recorded on video with a static and a mobile camera and independently rated by four examiners. Two of them were licensed paramedics, two were qualified emergency physicians. Each of the examiners had several years of experience in pre-hospital emergency medicine and they were all trained educators and instructors for both paramedics and physicians. As the GRS was designed as a self-explanatory and easy to use tool and in order to avoid any bias on interrater reliability, there were no preliminary briefings for the raters and their judgment had to be based solely on their professional expertise. Each team was evaluated as a unit by the raters and no conclusions on individual performances were drawn.

SPSS statistics software version 24.0.0.1 (IBM, Armonk, New York, USA) was used for statistical analysis. Due to the small sample size and non-normal distribution of some of the parameters non-parametric testing was used. All data are presented as median (minimum; maximum). The Kruskal-Wallis test was used for intergroup comparisons of the median ratings of each team. For post hoc analysis a Dunn-Bonferroni correction was carried out. The interrater reliability was tested with the Kendall’s coefficient of concordance W.

## Results

### Construct validity

The median score of the four student-guided teams was 22.75 points (17;30). The four paramedic-guided teams achieved a median of 31.25 points (21;35) and the four physician guided teams a median of 39.00 (32;47). Comparing all twelve teams, the GRS significantly discriminated between the different levels of training (Kruskal-Wallis *p*-value = 0.007).

**Fig. 3:**
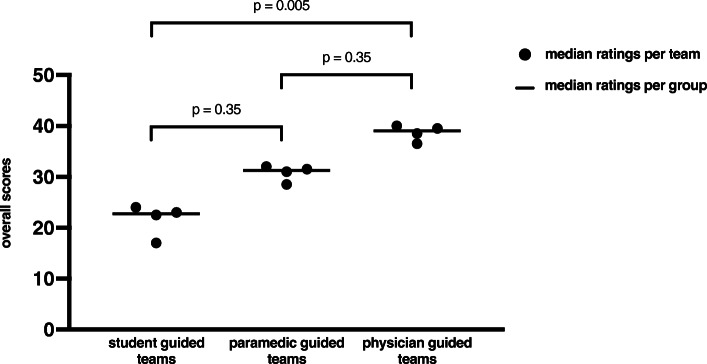
Graphical display of the overall results of the GRS. Median values of the overall scores of each group in the GRS are depicted. Median values of all four ratings per team that were used for further statistical analysis are also presented. P values are given for the Kruskal-Wallis-Test after Dunn-Bonferroni correction

Post hoc testing revealed statistical significance comparing student- and physician guided teams (*p* = 0.005), but not comparing students- and paramedics (*p* = 0.35) and paramedic- and physician guided teams (p = 0.35). The median values of all ratings per team and the detailed post-hoc analysis *p* values are illustrated in Fig. 3.

The overall rating scores in the TSC ranged from a median of 12 points (9;18) for student guided teams and a median of 16.75 (13;22) for paramedic guided teams. Physician guided teams scored a median of 16.50 (13;22). Similar to the GRS, the TSC showed significant discrimination between groups overall (Kruskal-Wallis *p*-value = 0.028). Post-hoc testing did not reach statistical significance (student- vs physician-guided teams (*p* = 0.052); student- vs paramedic guided teams (*p* = 0.076), paramedic- vs physician-guided teams (*p* = 1.00).

### Interrater-reliability

The interrater-reliability was measured with the Kendall’s coefficient of concordance W (Table 2). The Kendall’s coefficient of concordance W for the overall score in the GRS was 0.88. Moreover, in 9 of 10 items the concordance amongst examiners was high (0.64 to 0.90) in the GRS, only item 4 (patient’s position) yielded less consistent rating results (0.44). The highest concordance was achieved for item 8 (drug application), followed by item 7 (therapy and medication). For the items 2 (physical examination), 9 (patient safety overall), 10 (overall performance) a concordance coefficient of over 0.80 was reached.

**Table 2:** Kendall’s coefficient of concordance W for the GRS

Item	Item Name	Kendall’s W
1	Systematic assessment approach	0,75
2	Physical examination	0,86
3	Vital signs & Monitoring	0,70
4	Patient’s position	0,44
5	Temperature management	0,64
6	Handling of equipment	0,67
7	Therapy and medication	0,88
8	Drug application	0,90
9	Patient safety overall	0,82
10	Overall performance	0,84
Score	Overall Score in the GRS (all items)	0,88

Kendall’s W coefficient of concordance for the 10 items and the overall score of the GRS. Agreement amongst the 4 raters is high in 9 of 10 items and the overall score

In comparison, the TSC achieved a concordance of 0.84 in the overall score, yet for the single items the coefficient of concordance varied between 0.25 and 0.93.

## Discussion

The aim of the present study was to develop an assessment tool to objectively and reproducibly assess technical skills of trainees in emergency medicine simulation scenarios. A valid assessment and feedback guided by individual needs is critical to effective learning [29]. Previously established GRS in OSATS and objective structured clinical examinations (OSCE) formats in other fields of medicine proved to have a high construct validity and interrater reliability [19, 20, 22, 30]. Moreover, OSATS seems to be superior to other traditional methods of assessing clinical competencies [31].

In accordance to these findings the GRS in the present study significantly discriminated between novice (student-guided) and expert (physician-guided) simulation participants. The difference between the student guided teams and the paramedic-guided teams as well as the paramedic-guided teams and the physician-guided teams did not reach statistical significance in the post hoc analysis most likely due to the small sample size. The fact, that the GRS was able to discriminate between the groups although only the level of training and experience of the team leader varied between the groups while all other team members were 4th year medical students lacking professional experience and in light of the small sample size underlines the relevance of the results. Although students were well educated handling emergency medical scenarios, they generally lacked a sufficient amount of training in technical skills and practical experience in the field. In contrast, the paramedics could rely on numerous skill-trainings during their education and experience on duty on an ambulance. But as they usually rely on emergency physicians in the field to treat severely ill or injured patients, they encountered in part difficulties in clinical decision making and guideline conform therapy.

The TSC, used to cross check the results of the GRS showed a similar picture, but was incapable of distinguishing between paramedics and emergency physicians. In comparison to the GRS, differences in the performances of incorrectly done tasks are not further graded by the TSC, as the TSC only considers the final result of a task, i.e. either a correctly or incorrectly done task. In retrospective, a more detailed TSC might eventually have performed more precisely. However, to further analyze incorrect tasks by a TSC, an extensive TSC would be necessary most likely resulting in the loss of the “check list character”. In contrast, the GRS is capable of a more detailed rating of incorrectly or not completely accomplished tasks by the 5-point Likert scale. Therefore, it is possible to appreciate any actions performed during a task with the help of the GRS even if the whole task has to be considered as incomplete or incorrect. Hence, partially completed or moderately incorrect actions may result in a higher scoring and consequently in better discrimination between different teams. These findings are in line with previous studies preferring the GRS as primary or even stand-alone rating tool to assess technical skills as it is considered to be more reliable, appropriate and sensitive to the level of expertise [21, 22, 30, 32]. Nevertheless, a sophisticated and detailed TSC may add precise insights on shortcomings in the skill-set of a trainee.

A high interrater reliability could be demonstrated for the GRS in the present study, although no preliminary briefing of the rating team was performed. Neither any instruction on how to apply the rating tools, nor the precise definition of the single items of the GRS/TSC were given. Thus, any rater bias was avoided. These findings highlight that the GRS is an easy to use tool and due to the high standardization in emergency medicine with systematic approaches, guidelines, procedures and algorithms, agreement amid instructors is generally given. Further studies considering the GRS to be a time efficient and feasible tool [33, 34] support these results. Yet, even more consistent results might have been achieved with a preliminary briefing among the raters on how to use the tool or any objectives of the items of the GRS, as these may vary slightly in diverse simulation scenarios.

Despite a growing number of available rating tools, robust data on how to use objective structured assessment of technical skills to successfully improve learning and performance is lacking. Further research on the principles of learning and training effectiveness is needed, as well as evidence on transferring these achievements from the simulation environment into ultimately improved patient care.

## Limitations

The most important limitation of the present study represents the small sample size limiting statistical significance and generalizability. Thus, we consider the study as a pilot project requiring further evaluation and validation. Nevertheless, the present findings with significant discrimination of the GRS between teams despite the small sample size indicate relevant results that warrant further exploration. Due to the very small study cohort no randomization could be performed. Participants were allocated to the teams according to their self-reported level of training and experience in order to create comparable team members for every team leader.

With no pre-test of the real skillset and knowledge of each participant before the scenarios, the assignment to the different teams was completely based on the reported level of training and education. Especially in the teams led by a medical student or a paramedic, differences in the level of training and pre-hospital and emergency medical experience could not completely be ruled out. As all participants attended in their free time after work or after their curricular commitments, the authors consider a selection bias as well. To some degree, the examiners knew about the level of training of a participant beforehand and in some cases also had a deeper insight in their skillset from previous collaborations due to their work as clinicians or instructors. However, the GRS was used to assess the team as a whole, thus mitigating the effect of knowledge of individual skill sets of some of the participants.

Two of the raters were present during the scenario, recording, instructing and debriefing the simulation. They might have seen or heard additional information, which was not observable on the video clip for the other examiners. In order to minimize loss of information for the raters not present during simulation, a mobile camera was used in addition to a static one for acquisition of close ups and dynamic scene following.

## Conclusions

In the present study, a new GRS for OSATS in emergency medical simulation was developed and preliminarily validated. The GRS demonstrated a good discrimination between teams with different levels of expertise. Additionally, the GRS showed a high interrater reliability. Thus, the GRS represents a novel tool for the assessment of technical skills in emergency simulation for education and training purposes. Certainly, further research is mandatory to confirm the findings in larger cohorts with different skill levels, scenarios and settings (e.g. trauma or pediatric).

## Data Availability

The datasets used and/or analysed during the current study are available from the corresponding author on reasonable request.

## Abbreviations

ANTS: Anaesthetist’s non-technical skills assessment
AV: Atrioventricular
CRT: Capillary Refill Time
CI: Confidence Interval
ECG: Electrocardiogram
EMT: Emergency medical technician
F: Female
GRS: Global Rating Scale
M: Male
OSATS: Objective structured assessment of technical skills
OSCE: Objective structured clinical examination
QSATS: Queen’s Simulation Assessment Tool
TSC: Task Specific Checklist
